# Impact of Temozolomide on Immune Response during Malignant Glioma Chemotherapy

**DOI:** 10.1155/2012/831090

**Published:** 2012-10-24

**Authors:** Sadhak Sengupta, Jaclyn Marrinan, Caroline Frishman, Prakash Sampath

**Affiliations:** ^1^Brain Tumor Laboratory, Roger Williams Medical Center, 825 Chalkstone Avenue, Prior 222, Providence, RI 02908, USA; ^2^Department of Neurological Surgery, Boston University School of Medicine, Boston, MA 02118, USA

## Abstract

Malignant glioma, or glioblastoma, is the most common and lethal form of brain tumor with a median survival time of 15 months. The established therapeutic regimen includes a tripartite therapy of surgical resection followed by radiation and temozolomide (TMZ) chemotherapy, concurrently with radiation and then as an adjuvant. TMZ, a DNA alkylating agent, is the most successful antiglioma drug and has added several months to the life expectancy of malignant glioma patients. However, TMZ is also responsible for inducing lymphopenia and myelosuppression in malignant glioma patients undergoing chemotherapy. Although TMZ-induced lymphopenia has been attributed to facilitate antitumor vaccination studies by inducing passive immune response, in general lymphopenic conditions have been associated with poor immune surveillance leading to opportunistic infections in glioma patients, as well as disrupting active antiglioma immune response by depleting both T and NK cells. Deletion of O6-methylguanine-DNA-methyltransferase (MGMT) activity, a DNA repair enzyme, by temozolomide has been determined to be the cause of lymphopenia. Drug-resistant mutation of the MGMT protein has been shown to render chemoprotection against TMZ. The immune modulating role of TMZ during glioma chemotherapy and possible mechanisms to establish a strong TMZ-resistant immune response have been discussed.

## 1. Introduction

Glioblastoma, or malignant glioma (MG), is the most aggressive adult brain cancer and accounts for more than 50% of all glioma cases diagnosed [1]. Despite research efforts, the average lifespan for a MG patient postdiagnosis is 14.6 months with most patients experiencing tumor relapse and outgrowth within 7 months of initial radiation therapy [2–4]. For these reasons, MG care remains largely palliative. Brain tumors constitute a unique class of tumors among the most difficult to treat because they are anatomically shielded by the blood-brain barrier, lack a lymphatic drainage system, and grow in a strongly immunosuppressive environment due mainly to tumor infiltration by regulatory T cells (Tregs) marked with the rapid proliferation of tumor cells [5–8].

The overall prognosis of MG has increased only slightly over the past 30 years. High-grade glioma outcome is generally dependent on factors such as age, histology, and extent of surgical resection. Other predictors of survival tend to be the tumor size and proliferation index overexpression [9, 10]. Current treatment options include surgery, radiation, and chemotherapy [11]. Surgery combined with radiotherapy and chemotherapy has proven to prolong patient survival [2, 12]. However, patients diagnosed with MG who are treated with traditional therapeutic methods often experience harmful side effects like loss of cognitive process from surgery, inflammatory responses from chemotherapy or even induction of secondary cancers from radiation therapy [13]. Temozolomide (TMZ), an oral alkylating chemotherapeutic agent, is now a part of postresection standard chemotherapy for MG treatment [14]. Although, radiotherapy followed by concomitant and adjuvant TMZ therapy showed promising results in patient survival [2], recent studies indicate a rather disconcerting outcome. Over 40% of patients undergoing chemotherapy and 55% of newly diagnosed cases do not benefit at all from TMZ therapy [15, 16]. Further, a limitation in the established treatment protocol is the tumor's location within the brain [5, 17]. As a result, novel strategies are continually being tested to improve patient survival, quality of life, and overall outcomes. Targeted immunotherapy has received a great deal of interest in recent years [5, 18]. Its appeal is in being able to utilize a tumor-specific cytotoxic agent that preferentially targets tumor cells, without causing damage to the surrounding brain tissue. 

However, active immunotherapy in glioma patients faces a major roadblock when it is ready to be used for translational therapeutic purposes due to TMZ-induced lymphopenia [19, 20]. TMZ, like other chemotherapeutic drugs, comes with its own side effects that include moderate to severe lymphopenia or abnormally low levels of white blood cells. In this paper, we will discuss the role of TMZ during glioma chemotherapy and possible remedies to mitigate TMZ-induced lymphopenia during active MG immunotherapy.

## 2. Temozolomide

To date, the most widely used and effective chemotherapeutic drug for glioblastoma patients is temozolomide (TMZ). TMZ is a prodrug of the alkylating agent 5-(3 methyltriazen-1-yl) imidazole-4-carboximide (MTIC) [21]. Upon administration, TMZ spontaneously hydrolyzes at physiological pH to the active metabolite (MTIC). MTIC is then further hydrolyzed to the active methylating species, methylhydrazine [15, 22]. TMZ exerts its antitumor effects by methylating guanine at the N7 (70% of adducts) and O6 (5% of adducts) positions and adenine at the N3 position (9% of adducts) [22–24]. These adducts create DNA damage in tumor cells that result in apoptosis and cytotoxicity [25]. The base-excision repair mechanism repairs the N7 guanine and N3 adenine adducts; however, TMZ preferentially targets the middle guanine residue of GGG DNA sequences, which produces O6-methyl-guanine (O6MeG), the most potent killing adduct [26]. O6MeG preferentially pairs with thymine and leads to GC-AT transitions, beginning a cycle of DNA mismatch repair that leads to DNA double-stranded breaks and, ultimately, apoptosis of tumor cells [23].

A 2005 study by the European Organization for the Research and Treatment of Cancer and the National Cancer Institute of Canada Clinical Trials Group (EORTC/NCIC) launched TMZ as the new standard of care for MG when a significant improvement was demonstrated in both overall median survival (12.1 months in the standard radiotherapy cohort versus 14.6 months in the TMZ-treated cohort) and 2-year survival (10% versus 27%) [2]. These findings refuted long-held reservations regarding the widespread use and safety of TMZ. TMZ is currently licensed by FDA (in USA) and EMA (in EU) to treat MG concurrently with radiation, followed by an adjuvant maintenance period of TMZ treatment [19]. It is also licensed as a therapy for refractory anaplastic astrocytoma (in USA) as well as recurrent MG (in EU) and is approved for use in metastatic melanoma patients in over 20 countries [19]. A major benefit of TMZ is its ability to penetrate the blood-brain barrier, with almost 100% bioavailability. However, as the current standard of chemotherapeutic care, TMZ is associated with only 2.5 months of additional survival for patients when compared to treatment with radiation therapy alone [17]. In recent studies, it has been confirmed that patients with methylated promoter of DNA-repair enzyme O6-methylguanine-DNA methyltransferase (MGMT) fare significantly better in TMZ response with a median survival of 21.7 months [27]. 

## 3. MGMT and Its Role in TMZ Resistance

The most important prognostic factor for survival and predictive biomarker for TMZ response is the methylation status of MGMT. MGMT is a DNA repair protein that repairs the O6MeG lesion created by TMZ. MGMT irreversibly binds to and degrades O6MeG adducts from DNA [26, 28]. MGMT encodes human alkyl guanine transferase, which transfers the methyl group from O6MeG to an internal cysteine residue, simultaneously repairing DNA and counteracting the cytotoxicity of TMZ and other alkylating agents [25, 29–31]. When O6MeG is repaired by MGMT, cells become either resistant to O6MeG-triggered apoptosis or die due to *N*-alkylation lesions like 3-methyladenine, 3-methylguanine, and apurinic sites created as side products of *N*-methylpurine hydrolysis. Depletion of MGMT by O6-benzylguanine (O6-BG, a specific MGMT inhibitor) prevents TMZ-induced apoptosis [26]. It has been widely documented that the level of MGMT activity is correlated with tumor cell resistance to the methylating chemotherapeutic drugs [32–37]. Kitange et al. compared five primary xenograft models of TMZ resistance and found significant upregulation of MGMT expression in resistant MG xenograft lines, where MGMT was proven mechanistically linked to TMZ resistance. O6-BG restored TMZ sensitivity only in those lines that overexpressed MGMT [38]. Epigenetic silencing by promoter methylation (reviewed by Riemenschneider et al. [39]) and MGMT depletion [26, 40] provides relief from TMZ resistance in MG. 

## 4. Role of TMZ in Inducing Lymphopenia and Its Role Immune Response during Glioma Chemotherapy

While lower expression of MGMT is desired in glioma cells for better TMZ efficacy, successful immune response in MG patients depends upon the functional efficiency of MGMT in conferring resistance to TMZ cytotoxicity. Although TMZ has proven to be generally well tolerated among patients, it is known to induce lymphopenia or abnormally low levels of white blood cells. An initial report of TMZ-induced lymphopenia was reported by Brock et al. [41] and has been confirmed by others in recent years [42–46]. Bone marrow cells are particularly vulnerable to TMZ due to lower MGMT activity than tumors, resulting in myelosuppression [23, 31, 47].

It has been observed that TMZ selectively targets the human monocytes but not dendritic cells that are derived from these monocytes [48]. MGMT levels decline during maturation of monocytes into dendritic cells (DCs) [49] which was contradictory to the established fact that low MGMT levels were required for TMZ sensitivity. The concept of selective affinity of TMZ towards monocytes was further confirmed in an animal experiment where either GL261 mouse glioma cells in a syngeneic model or U87 human glioma in a xenograft model were treated with systemic application of anti-CCL2 monoclonal antibody (MAb). Cotreatment of these animals with TMZ significantly prolonged their survival (8/10) [50]. Malignant glioma secretes CCL2 or monocyte chemoattractant protein-1 (MCP-1) to stimulate monocytes to migrate to the tumor site where they are converted into immunosuppressive tumor-associated macrophages and myeloid-derived suppressor cells (MDSCs) and facilitates tumor growth [51–53]. While anti-CCL2 MAb prevented the migration of the monocytes to the tumor site, TMZ probably played a role in surveillance and killing of monocytes that reached the tumor site, thereby mitigating immune suppression. In a recent report, Bauer et al. have attributed the selective apoptosis of monocytes by TMZ to ATM/ATR pathway-induced p53 activation, rather than any active role played by MGMT [54]. In another recent study, peripheral blood mononuclear cells (PBMCs) of 25 MG patients were tested for immune modulation prior to and 4 weeks after radiation therapy-temozolomide (RT-TMZ) treatment [55]. The authors observed measurable decrease in tumor-infiltrating CD8 CTLs and NK cells and proportional increase in functional Tregs. Further, they also observed that monocytes retained their ability to differentiate into DCs after RT-TMZ treatment. Yet again, in an animal model study by Banissi et al., low-dose metronomic administration of TMZ, but not standard regimen, demonstrated a decrease in circulating Tregs in a glioma rat model [56]. Results from this study were completely opposite to the above-discussed human study but corroborated with earlier findings of pan-CD4^+^ lymphopenia in TMZ-treated melanoma patients [57]. Blockade of CD25 or IL2-receptor alpha chain by monoclonal antibodies during TMZ-induced lymphopenia selectively abrogated Tregs in mice and humans and correlated with enhanced immunity in patients with MG [58, 59]. The Treg reduction was associated with a significant expansion of vaccine-stimulated antitumor effector T cells. Several studies in both mice [60] and humans [61, 62] have shown that it may be possible to exploit this timeframe of lymphopenia in order to amplify immune response since lymphodepletion has been shown to decrease competition at the surface of antigen-presenting cells, therefore improving cytokine availability, which, in turn, amplifies T-cell activity and depletes Tregs [44]. Reardon and his colleagues have succinctly remarked that lymphodepletion resets host's immune system and thus eliminates host's tolerance to autologous tumor antigens. Efficacy of antitumor vaccines can thus be enhanced by vaccination immediately after lymphodepleting chemotherapy [63]. In as study with glioma patients, who were vaccinated with autologous tumor-lysate-loaded DCs after RT-TMZ therapy, increased frequency of interferon-gamma-positive CD4^+^ T cells was observed [64]. TMZ chemotherapy also increased cross-priming of tumor antigen-specific CD4^+^ and CD8^+^ T cells in an animal model and suppressed Treg activation when TMZ-treated animals were vaccinated with tumor-antigen pulsed DCs [65].

However, other studies have sharply contradicted the aforementioned optimistic views. In a recent study performed on pathologically verified malignant glioma patients who were treated with focal radiotherapy plus concomitant daily TMZ, patients often suffered from severe lymphopenia, analyzed by assessing adverse effects including decrease in white blood cell counts, lymphocyte counts, and neutrocyte counts. Eighty-two percent of the patients suffered one or more adverse effects; lymphopenia (68%) was the most frequent adverse effect, with 32% of patients suffering CTC grade 4 lymphopenia [66]. The significant and long-lasting lymphopenia induced by TMZ is the biggest concern associated with its use. Preferential CD4^+^ lymphopenia has been observed in melanoma patients that were treated with an extended dose of TMZ [57]. These patients were also reported to have a high incidence of opportunistic infections. In a similar study performed on children and adolescents with brain tumors, improvement in survival was also associated with a higher rate of virus infections [67]. Similarly, concomitant radiotherapy with TMZ increased the proportion of functional Tregs while depleting the incidence of NK cells in MG patients, suggesting an increased immunosuppressive environment [55]. In another recent study, a combined immunotherapy of mTOR suppression and TMZ administration resulted in robust depletion of CD4^+^, CD8^+^, NK, and B cells leading to increased opportunistic infections in the treated group [68].

Depletion of O6-alkylguanine-DNA alkyl transferase (AT/MGMT) activity in PBMCs of TMZ-treated patients has been directly linked to the cause of myelosuppression [47]. In this study, however, TMZ was sequentially administered with fotemustine. Fotemustine is a nitrosourea alkylating agent and uses the same working principle as TMZ. D'Atri et al. corroborated this result by showing that TMZ induced a rapid depletion of MGMT activity in a concentration-dependent manner [69].

## 5. P140KMGMT and Chemoprotection**** against TMZ

Overexpression of MGMT has not been successful in resisting TMZ cytotoxicity [47, 69]. This was also confirmed by Briegert et al. where they showed that high levels of MGMT did not confer human monocytes resistant to alkylating chemotherapy [49]. Transfer of drug resistance genes to hematopoietic stem cells offers the potential to protect cancer patients from drug-induced myelosuppression and to increase the number of gene-modified cells by *in vivo* selection [70]. Recent studies have shown promising effects of chemoprotection in hematopoietic cells by mutating the proline residue at 140 of the MGMT peptide to lysine (P140KMGMT). Introduction of other coding region polymorphisms like L84F has been shown to alter the stability of target cells rather than affecting TMZ sensitivity [71]. Mice transplanted with P140KMGMT-transduced cells showed significant resistance to the myelosuppressive effects of TMZ and O6-BG. Following drug treatment, transduced cells were seen in all peripheral blood lineages, and secondary transplant experiments proved that selection had occurred at the stem cell level [70]. Zielske et al. showed that myelosuppression can be mitigated by P140KMGMT gene transfer into hematopoietic stem cells and bone marrow transplantation in order to continue with TMZ dose escalation [72]. NOD/SCID mice xenotransplanted with P140KMGMT-overexpressing human CD34^+^ cells conferred chemoresistance against O6-BG and O6-BG and 1,3-bis(2-chloroethyl)-1-nitrosourea (BCNU) [73]. Overexpression of P140KMGMT increased the DNA-repair capacity of both primary human CD34^+^ cells and K562 cell lines [23]. Using a canine model, Neff et al. have shown that P140KMGMT-transfected CD34^+^ hematopoietic cells prevented myelosuppression after repeated treatment with TMZ and O6-BG [74]. Chemoprotection was also observed in dogs engrafted with P140KMGMT-transfected CD34^+^ cells when challenged with O6-BG and BCNU [75]. P140KMGMT-based gene therapy developed significant resistance to TMZ in immunocompetent NK-92 and TALL-104 cells and protected their ability to kill K562 leukemia cells. The modified cells developed significant resistance to TMZ compared to nonmodified cells, and genetic modification of these cells did not affect their ability to kill K562 cells [76]. Simultaneous overexpression of multidrug resistance 1 (MDR1) and P140KMGMT in CD34^+^ cells resulted in stable populations of MGMT^+^ cells upon exposure to O6BG/TMZ therapy [77].

## 6. Future Directions

Targeted immunotherapy of MG using antigen-redirected chimeric T cells is one of the mechanisms of active immunotherapy of MG that has been successfully developed in recent years [78–83]. This approach of MG immunotherapy may be functionally precise but may be inhibited by concurrent myelosuppressive TMZ, which produces a survival benefit in MG [20]. TMZ-induced immunosuppression also leads to opportunistic infections in MG patients undergoing chemotherapy (reviewed by Kizilarslanoglu et al. [84]) [57, 67]. As evidenced from the available literature, P140KMGMT mutation is successful in blocking the TMZ-induced cytotoxic depletion of MGMT activity in transduced hematological cells. However, P140KMGMT mutation has yet to be used for the treatment of immunosuppressive side effects of TMZ chemotherapy in MG patients. Autologous T cells from MG patients currently undergoing chemotherapy can be genetically modified to express P140KMGMT and render them resistant to TMZ. This will have two major benefits. On one hand these cells will be viable against chemotherapeutic cytotoxicity. On the other hand, these viable cells will be efficient in establishing a strong immune response because of, and eventually overcoming the huge immune void eventually created by TMZ-induced lymphopenia.
